# The N-Terminal Domain of LIC12756 Is the Key Determinant of This Protein’s Anti-Sigma Activity Toward LIC12757 in Pathogenic *Leptospira interrogans*

**DOI:** 10.3390/pathogens15040379

**Published:** 2026-04-01

**Authors:** Sabina Kędzierska-Mieszkowska

**Affiliations:** Department of General and Medical Biochemistry, Faculty of Biology, University of Gdańsk, 80-308 Gdańsk, Poland; sabina.kedzierska-mieszkowska@ug.edu.pl; Tel./Fax: +48-58-523-6064

**Keywords:** *Leptospira interrogans*, ECF sigma factor, anti-sigma factor, protein–protein interaction, transcriptional regulation

## Abstract

Extracytoplasmic function (ECF) σ factors are central regulators of bacterial adaptation to environmental changes. The genome of *Leptospira interrogans* encodes 11 such factors, including LIC12757. Previous studies have shown that LIC12757 is regulated by the FecR-like protein LIC12756, forming a regulatory system similar to the *Escherichia coli* FecI-FecR system. Here, the domain-specific regulatory role of LIC12756 was investigated. Interactions between LIC12757 and several LIC12756 variants, including the N-terminal domain (NTD) alone, NTD with half or full transmembrane domain (NTD-TMD), and full-length LIC12756 (FL, control), were analyzed using the BACTH system. During logarithmic growth, interactions were detected only with FL and NTD-TMD, whereas in the stationary phase, all variants interacted with varying strengths. Pull-down assays using His_6_-tagged NTD confirmed its direct binding to LIC12757. Promoter activity analysis revealed that the NTD alone functions as an anti-σ factor in the logarithmic stage of growth. However, it is insufficient for full activation of LIC12757-dependent transcription during the stationary phase, as observed with FL protein. The NTD-TMD variant caused only minor stimulation compared to FL. These results indicate that NTD is a key determinant of LIC12756’s anti-σ activity toward LIC12757, whereas full activation of LIC12757 requires additional extrinsic signals, which are likely sensed by the C-terminal extracytoplasmic region (ECR). These findings provide mechanistic insight into ECF σ factor regulation in *L. interrogans*.

## 1. Introduction

Bacterial sigma (σ) factors are essential transcriptional regulators that direct RNA polymerase to specific promoters, enabling rapid reprogramming of gene expression in response to environmental and host-derived signals [1,2,3,4,5]. These factors can be broadly divided into primary and alternative σ factors, which differ in their roles in basal transcription and adaptive responses [2,6]. Alternative σ factors play a central role in bacterial adaptation by orchestrating global transcriptional responses to changing conditions. Among them, extracytoplasmic function σ factors (ECFs) constitute one of the largest and most diverse subfamilies of the σ^70^ family, which is primarily involved in coordinating adaptive responses to envelope stress, nutrient limitation, and virulence-associated processes [1,6,7,8,9,10,11,12,13,14,15]. ECFs typically control relatively narrow and specialized regulons, allowing precise and stimulus-specific transcriptional responses. The activity of ECFs is primarily regulated by cognate anti-σ factors (transmembrane or soluble proteins), which bind their partner σ factor and prevent its association with the RNA polymerase core enzyme under non-inducing conditions [1,6,8,10,16]. Upon environmental or physiological signals, ECFs are released through three major mechanisms: (1) direct environmental sensing, in which the anti-σ factor undergoes conformational changes in response to extracellular or redox signals; (2) regulated proteolysis (RIP), where membrane-bound anti-σ proteins are sequentially cleaved to liberate the σ factor; and (3) partner-switching, in which interactions with additional regulatory proteins modulate the anti-σ/σ complex [6]. These diverse regulatory strategies enable ECFs to control specialized regulons and fine-tune adaptive responses, ensuring bacterial survival under a wide range of stress conditions [8,16]. On the other hand, the well-known FecI-FecR regulatory system from *E. coli*, involved in iron transport [17,18,19], differs from the typical σ/anti-σ factor model [10]. The membrane-anchored FecR not only suppresses its partner σ factor FecI in the absence of ferric citrate, but is also essential for FecI full transcriptional activation in response to iron-limiting conditions [10]. Furthermore, FecR appears to stabilize FecI, as FecI is unstable in the absence of FecR [10]. Recent studies demonstrated that the intramembrane protease RseP mediates proteolytic processing of FecR, enabling efficient transmission of ferric citrate signals to FecI and underscoring the pivotal role of regulated proteolysis in ECF σ factor control [20,21].

Pathogenic *L. interrogans*, the causative agent of leptospirosis—a globally distributed zoonotic disease—encounters diverse environments throughout its life cycle, ranging from environmental water reservoirs to mammalian hosts [22,23,24,25]. This requires dynamic regulation of its gene expression to ensure rapid adaptation to changing conditions. Similarly to other bacteria, *L. interrogans* utilizes regulatory proteins, including σ factors, to coordinate this process at the transcriptional level. However, the mechanisms governing transcriptional regulation in this pathogen remain poorly characterized. It is worth noting that leptospirosis is a disease with a significant public health impact, causing over one million severe cases and approximately 60,000 deaths annually, mainly in tropical and subtropical regions [26]. Leptospiral infection occurs through contact with urine from infected animals or via contaminated water and soil. Clinical manifestations vary from mild, flu-like symptoms to severe, potentially fatal disease with multi-organ involvement [22,27,28]. Beyond its impact on human health, it also contributes to substantial economic losses in livestock production by affecting animal health, reproduction, and productivity [29,30,31]. These facts highlight the need to better understand the regulatory networks, including ECFs, that enable *L. interrogans* to adapt to diverse environments and host-induced stresses. Such knowledge is crucial for understanding the pathogenesis of leptospirosis and for the development of effective disease control strategies.

Recent comparative genomics and in silico genome-wide analyses have revealed that the *L. interrogans* genome encodes 11 predicted ECFs. Interestingly, it lacks canonical stress-responsive σ factors such as RpoS (σ^38^) and RpoH (σ^32^), suggesting that ECF pathways may serve as central regulators of both environmental adaptation and pathogenicity [32,33]. Despite their predicted importance, most ECFs in this pathogen have yet to be fully characterized, and the mechanisms by which they control gene expression are still largely unexplored. Among these factors, LIC12757 has been shown to function as an ECF σ factor [34]. Recent studies have shown that its activity is regulated by a putative FecR-like regulator, LIC12756, via protein–protein interactions [35]. Importantly, LIC12756 appears to function as a dual regulator of LIC12757, acting both as an inhibitor (anti-σ) and as a positive regulator in response to specific environmental signals, including nutrient-limiting conditions, such as iron deficiency [35]. Consequently, it may also be involved in responses to host-induced stress. The dual functionality of LIC12756 suggests that, unlike typical anti-σ factors, it is a non-canonical regulatory protein with FecR-like characteristics. Its domain organization resembles that of FecR and consists of distinct N-terminal cytoplasmic (NTD), transmembrane (TMD), and C-terminal periplasmic (ECR) regions, indicating its possible role in signal transduction [35]. Based on these observations, it is hypothesized that LIC12756 regulates LIC12757 through distinct functional domains that mediate inhibitory or activating interactions, depending on environmental conditions. To test this hypothesis, domain-specific interactions between LIC12756 variants and LIC12757 were examined using a bacterial adenylate cyclase two-hybrid (BACTH) system, and the resulting functional effects were assessed using an in vivo promoter activity assay. These analyses provide mechanistic insights into LIC12756 function, demonstrating that its NTD primarily mediates the anti-σ activity toward LIC12757, whereas the full-length protein is required for transcriptional activation, possibly reflecting a need for proteolytic processing that is analogous to the *E. coli* FecR regulator [21,22].

## 2. Materials and Methods

### 2.1. Bacterial Strains, Growth Media, Plasmids, and Genomic DNA

**Figure 1:**
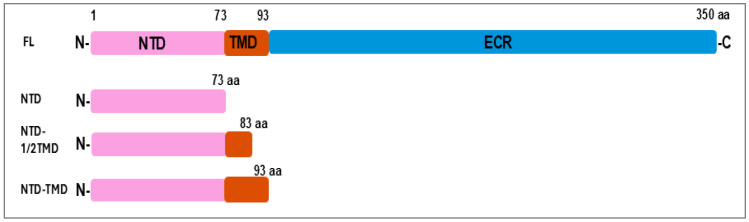
Schematic representation of LIC12756 domain organization and its variants used in this study (based on [35]). The full-length protein consists of a cytoplasmic N-terminal domain (NTD; 1–73 aa, pink), a transmembrane helical domain (TMD; 74–93 aa, orange), and a periplasmic C-terminal domain/extracytoplasmic region (CTD/ECR; 94–350 aa, blue). Variants of LIC12756 used in this study are indicated below the full-length protein.

*E. coli* strains and plasmids used in this study are listed in Table 1. *E. coli* DH5α was used for cloning of *LIC12756* variants (Figure 1) into the pJET1.2 vector, while *E. coli* XL1-Blue was used for subsequent subcloning into BACTH system plasmids (Euromedex, Souffelweyersheim, France). The *E. coli* MG1655 strain was used for luciferase/promoter activity assay. The *cyaA*-deficient *E. coli* DHM1 strain was used as a host for detection of LIC12757-LIC12756 variant interactions (the BACTH assay). *E. coli* strains were routinely grown in LB medium or on LB agar at 30 or 37 °C. Media were supplemented with appropriate antibiotics at the following concentrations: ampicillin (100 μg/mL), kanamycin (30–50 μg/mL) and chloramphenicol (40 μg/mL). Protein complementation was assessed on LB agar plates supplemented with X-gal (Sigma-Aldrch, St. Louis, MO, USA), prepared according to the manufacturer’s instructions (Euromedex, Souffelweyersheim, France). Expression of hybrid proteins (T25-LIC12757 and T18-LIC12756 variants) was induced by adding IPTG to a final concentration of 0.5 mM.

Genomic DNA of *L. interrogans* serovar Copenhageni, previously described [36], was used as a template for PCR amplification of LIC12756 variants. The amplified fragments were initially cloned into the pJET1.2/blunt vector and subsequently subcloned into BACTH system plasmids.

Nucleotide sequence of the *L. interrogans LIC12756* gene was obtained from the complete genome sequence of *L. interrogans* serovar Copenhageni strain Fiocruz L1-130 deposited in GenBank under accession number AE0168823.1 (chromosome I) [37].

**Table 1:** *E. coli* strains and plasmids used in this study.

*E. coli* Strain/Plasmid	Genotype	Reference/Source
DH5α	*supE44*, *hsdR1*, *recA1*, *endA1*, *gyrA1*, *gyrA96*, *thi-1*, *relA1*	laboratory stock
DHM1	*F*-, *cya-854*, *recA1, endA1*, *gyrA96 (Nal r), thi1, hsdR17, spoT1, rfbD1, glnV44(AS)*	Euromedex (France)
MG1655	*F*-, lambda^−^ *ilvG^−^, rfb*-50, *rph*-1	laboratory stock
XL1-Blue	*recA1, endA1, gyrA96, thi-1 hsdR17, supE44, relA1, lac [F’proAB lacIq Z∆M15Tn10(Tetr)]*	laboratory stock
BL21(λDE3)	*F^−^ ompT gal dcm lon hsdS_B_(r_B_^−^m_B_^−^) λ(DE3 [lacI lacUV5-T7p07 ind1 sam7 nin5]) [malB^+^]_K-12_(λ^S^)*	Novagen
pJET1.2/blunt	PCR cloning vector carrying an ampicillin resistance gene	ThermoFisher Scientific, Poland
pKT25	a pSU40-derived plasmid encoding the T25 fragment of CyaA (1–224 aa) under control of the *lac* promoter, carrying a kanamycin resistance gene	Euromedex (France)
pUT18C	a pUC19-derived plasmid encoding the T18 fragment of CyaA (225–399 aa) under control of the *lac* promoter, carrying an ampicillin resistance gene	Euromedex (France)
pKT25-zip	pKT25 derivative encoding GCN4-T25 fusion protein, used with pUT18C-zip as a positive control (BACTH assay)	Euromedex (France)
pUT18C-zip	pUT18C derivative encoding GCN4-T18 fusion protein, used with pKT25-zip as a positive control (BACTH assay)	Euromedex (France)
pAC-LIC12757	pAC7 derivative containing the *L. interrogans LIC_12757* gene under control of the *pBAD* promoter and a chloramphenicol resistance gene; used for luciferase activity assay	[38]
prLIC12757luxAB	pGB2 derivative containing the *V. harveyi luxAB* genes and a promoter region of the *L. interrogans LIC_12757* gene (used for luciferase activity assay)	[34]
pKT25-LIC12757	pKT25 derivative encoding a fusion protein T25-LIC12757 (BACTH assay)	[35]
pUT18C-LIC12756	pUT18C derivative encoding a fusion protein T18-LIC_12756 (BACTH assay)	[35]
pUT18-NTD LIC12756	pUT18C derivative encoding a fusion protein T18-NTD LIC12756 (BACTH assay)	this study
pUT18C-NTD ½ TMD LIC12756	pUT18C derivative encoding a fusion protein T18-NTD ½ TMD LIC_12756 (BACTH assay)	this study
pUT18C-NTD TMD LIC12767	pUT18C derivative encoding a fusion protein T18-NTD ½ TMD LIC_12756 (BACTH assay)	this study
pAC7	plasmid carrying *pBAD* promoter and chloramphenicol resistance gene	[39]
pAC-LIC12757-NTD LIC12756	pAC7 derivative carrying *LIC12757* and a 219 bp fragment of *LIC12756* under control of the *pBAD* promoter	this study

### 2.2. Plasmid Construction and DNA Manipulations

All plasmid constructs used in this study were generated by PCR amplification of the respective DNA fragments from *L. interrogans* genomic DNA using primers listed in Table 2 and Pfu DNA polymerase (EURx, Gdańsk, Poland). PCR reactions were performed as described previously [35]. The PCR products were evaluated by agarose gel electrophoresis, purified using a PCR Clean-up kit (A&A Biotechnology, Gdańsk, Poland), and initially cloned into the pJET1.2/blunt vector (see Table 1). Fragments of interest were excised from pJET1.2 by digestion with the appropriate restriction enzymes (EURx, Gdańsk, Poland) and subcloned into the final expression vectors, including pUT18C (carrying 219, 249, and 279 bp fragments of LIC12756) for BACTH assays, and pET28 (carrying the His-tagged N-terminal domain of LIC12756) for protein production. Additionally, LIC12757, together with a 219 bp fragment of *LIC12756* encoding its NTD, was cloned into pAC7 for an in vivo promoter activity assay to verify whether the presence of the CyaA T18 fragment affected the functionality of the truncated LIC12756 variants.

All plasmids were verified by DNA sequencing (Genomed S.A., Warsaw, Poland), and plasmid preparation and transformation of *E. coli* were carried out according to standard procedures [40].

### 2.3. Interaction Analysis of LIC_12757 and LIC_12756 Variants

#### 2.3.1. BACTH Assay—Screening and β-Galactosidase Activity Assays

A BACTH system kit (Euromedex, Souffelweyersheim, France) [41,42] was used to study protein–protein interactions between LIC_12757 and LIC_12756 variants according to the manufacturer’s instructions. *E. coli* DHM1(Δ*cyaA*) cells were co-transformed with plasmids pUT18C-LIC_12756 variants and pKT25-LIC_12757, as well as control plasmids (pKT25-zip, pUT18-zip, and plasmids pKT25-LIC12757 and pUT18C), and then selected on LB agar supplemented with ampicillin (100 μg/mL) and kanamycin (50 µg/mL). Several individual colonies were selected and grown at 30 °C overnight on LB-X-gal indicator plates containing the above-mentioned antibiotics and IPTG (0.5 mM). For β-galactosidase activity assay, the selected colonies were inoculated in LB medium supplemented with ampicillin, kanamycin and IPTG (0.5 mM) and incubated at 30 °C with vigorous agitation for 6 h (log phase of growth) or overnight (stationary phase). β-galactosidase activity assay was performed as described previously [35] and according to Miller’s method [43]. The enzyme activity was expressed in Miller units (1000 × OD_420_/OD_600_ of culture per mL of bacterial culture per min of reaction). Three independent experiments were performed, each with duplicate cultures.

#### 2.3.2. Affinity Pull-Down Assay of LIC12756 N-Terminal Domain with LIC12757

Two bacterial strains were used for this assay: *E. coli* BL21(λDE3) transformed with a pET28 vector carrying a 219 bp fragment of the *LIC12756* gene corresponding to its N-terminal domain (NTD; 73 aa), and *E. coli* MG1655 cells overproducing LIC12757 from pAC-LIC12757 [38].

*E. coli* BL21(λDE3) cells overproducing His_6_-tagged NTD were grown in LB medium (10 mL) to an OD_600_ ~ 0.6, and production of NTD was induced by the addition of IPTG (0.4 mM) for 3 h at 37 °C. *E. coli* MG1655 cells producing LIC12757 were grown in LB medium to an OD_600_ ~ 0.5, and expression of *LIC12757* was induced by the addition of 0.02% arabinose for 3 h at 37 °C. Next, cells were harvested by centrifugation and lysed using CelLyti B Cell Lysis Reagent (Sigma-Aldrich, St. Louis, MO, USA), diluted 1:5 in 20 mM Tris-HCl buffer (pH 8.0) supplemented with lysozyme (0.2 mg/mL) and DNase I (5 µg/mL), according to the manufacturer’s protocol. The lysate containing His_6_-tagged NTD (75 μL) was immobilized on 15 µL of Ni-NTA agarose (Macherey-Nagel, Düren, Germany) and washed five times with buffer A (20 mM Tris-HCl, pH 8.0, 300 mM NaCl, 20 mM imidazole). Subsequently, the LIC12757-containing lysate was added to the agarose-bound His_6_-tagged NTD, and binding was carried out at 4 °C. After a 2 h incubation, the agarose beads were washed extensively with buffer A containing 20 mM imidazole to remove non-specifically bound proteins. Proteins bound to NTD were eluted with buffer A containing 250 mM imidazole and analyzed by SDS–PAGE, followed by Western blotting using an anti-LIC12757 antibody [35].

Of note, the binding efficiency of His_6_-tagged NTD to Ni-NTA agarose beads was verified by a Coomassie-stained gel (Figure S1A) and Western blotting using an anti-His tag antibody (Cell Signaling Technology, Danvers, MA, USA) in a parallel sample prepared under the same conditions (Figure S1B). Agarose beads without immobilized NTD were incubated with the LIC12757-containing lysate and used as a background control sample to assess potential non-specific binding of LIC12757 to the nickel resin. Equal volumes of the same lysate (100 µL per sample) were used for both the NTD–LIC12758 interaction assay and the background control, ensuring comparable input material.

### 2.4. In Vivo Promoter Activity Assay—Luciferase Activity Measurement

Promoter activity was assessed in vivo according to previously described procedures [34,35,38] with minor modifications. *E. coli* MG1655 cultures harboring (1) the prLIC12757luxAB transcriptional fusion, (2) a two-plasmid system consisting of prLIC12757luxAB and pAC-LIC12757, and (3) the same two-plasmid system in combination with pUT18C carrying different *LIC12756* variants were grown at 30 °C in LB supplemented with appropriate antibiotics and IPTG (0.5 mM; induction of LIC12756 variants to OD_600_ ~ 0.45). Subsequently, *LIC12757* expression was induced by the addition of 0.02% arabinose for 6 h. Bacterial samples were collected during the log and stationary phases of growth to determine bacterial luciferase activity. For this purpose, 200 µL aliquots of culture were mixed with 7 µL of 10% *n*-decanal (Sigma/Merck KGaA, Darmstadt, Germany) in 96% ethanol, and light emission was monitored within 1 min using a luminometer (Berthold Technologies, Junior, Bad Wildbad, Germany). The luciferase activity was expressed in relative light units (RLU) divided by the optical density (OD_600_). Three independent experiments, with duplicate cultures, were carried out.

### 2.5. SDS-PAGE and Western Blot Analysis

Proteins were separated on 15% polyacrylamide gels using the Laemmli system and transferred to a nitrocellulose membrane for chemiluminescent detection as previously described [35]. LIC12757 was probed with anti-LIC12757 serum (1:500) [35], followed by HRP-conjugated goat anti-rabbit IgG (1:3000; Abcam, Cambridge, UK). The blot was then developed using BioVision ECF substrates (Gentaur, Sopot, Poland) and imaged using the Azure imaging system (Azure Biosystems, Dublin, CA, USA).

### 2.6. Statistical Analysis

Statistical analyses and graph generation were performed using GraphPad Prism version 10. Data normality was assessed by the Shapiro–Wilk test. Statistical significance was determined using an unpaired two-tailed *t*-test with Welch’s correction.

## 3. Results

### 3.1. The Role of LIC12756 Domains in Interactions with the ECF σ Factor LIC12757

**Figure 2:**
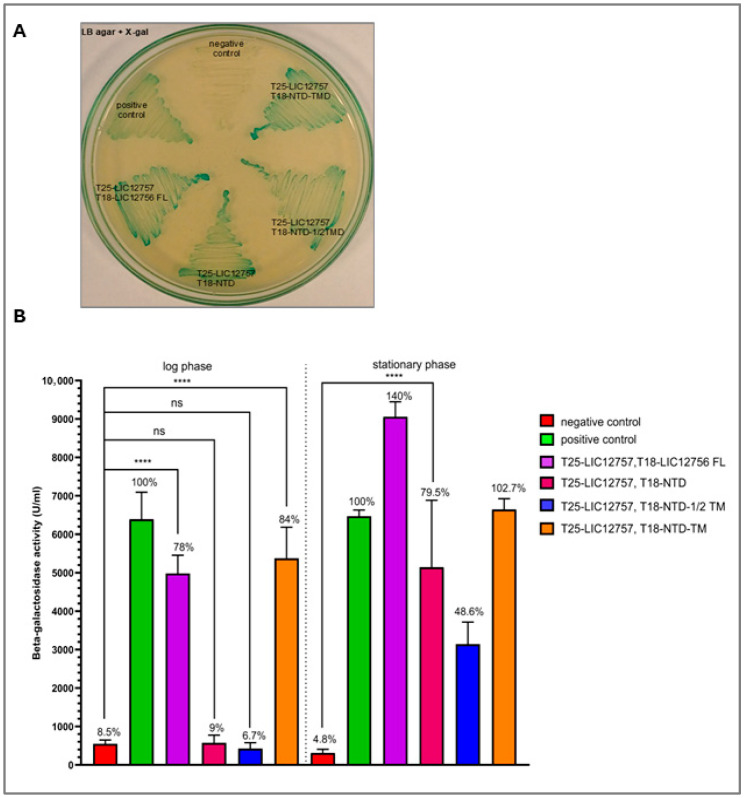
Analysis of interactions between LIC12756 variants and LIC12757 using the BACTH system. This assay was carried out using *E. coli* DHM1 cells co-transformed with the following plasmids: (1) pUT18C and pKT25-LIC12757, serving as a negative control; (2) the control plasmids pKT25-zip and pUT18C-zip (positive control); (3) pKT25-LIC12757 and pUT18C-LIC12756 variants (FL, NTD, NTD-1/2TMD, NTD-TMD). (**A**) Screening assay of *E. coli* DHM1 cells co-transformed with the above plasmids on an LB agar-X-gal indicator plate after 36 h of incubation at 30 °C. Blue colonies indicate protein–protein interaction, while colorless colonies indicate no detectable interaction. (**B**) Quantitative analysis of interactions between LIC12756 variants and LIC12757 based on β-galactosidase activity measured according to Miller’s method. Enzymatic activity is reported in Miller units. Interaction strength is presented as the percentage relative to the positive control (cells carrying pKT25-zip and pUT18C-zip, set as 100%), and the corresponding values are indicated above each bar. Measurements were performed during the log (6 h) and stationary (overnight) phases. Results represent the mean of three independent experiments, each performed with duplicate cultures, with standard deviations indicated. Statistical significance was assessed using an unpaired two-tailed *t*-test with Welch’s correction (GraphPad Prism 10): **** *p* < 0.0001; ns = not significant. In the log phase, interactions of NTD and NTD-1/2TMD with LIC12757 were not significantly different from the negative control. In the stationary phase, interactions of FL LIC12756, NTD, and NTD-TMD with LIC12757 were highly significant (*p* < 0.0001), while NTD-1/2TMD also displayed a significant interaction at a lower level (*p* = 0.0022); for clarity, only comparison with the NTD alone is shown. The mean β-galactosidase activity values for all variants and controls are provided in Supplementary Table S1, and exact *p*-values for all comparisons are provided in Supplementary Table S2. The dashed line indicates the log–stationary phase boundary.

To investigate the role of specific LIC12756 domains in the regulation of the ECF σ factor LIC12757 via protein–protein interactions using the BACTH system, a series of LIC12756 variants was generated using standard molecular biology methods (see Section 2.2 for details), including the cytoplasmic N-terminal domain (NTD), NTD with half of the transmembrane domain (NTD-1/2TMD), NTD with the full transmembrane domain (NTD-TMD), and full-length LIC12756 (FL) (Figure 1). Each variant of LIC12756 was fused to the T18 domain of adenylate cyclase (pUT18C) and co-produced with T25-LIC12757 (pKT25) in the BACTH system using *E. coli* DHM1 as the host. A screening assay conducted for 36 h at 30 °C demonstrated that all LIC12756 variants interacted with LIC12757 as judged by the indicator plate. As shown in Figure 2A, cells producing both fusion proteins (T18-LIC12756 variants and T25-LIC12757), as well as cells expressing the positive control plasmids (pKT25-zip and pUT18C-zip), formed blue colonies on LB agar-X-gal medium. In contrast, cells carrying the empty pUT18C and pKT25-LIC12757 (negative control) remained colorless, confirming the specificity of the observed interactions. Subsequently, β-galactosidase activity was measured to compare the strength of interactions between LIC12756 variants and LIC12757 during both log and stationary growth (Figure 2B). During logarithmic growth, strong interactions with LIC12757 were observed only for NTD-TMD (84%) and FL LIC12756 (78%) relative to the negative control (8.5%). In contrast, interactions of the NTD alone (9%) and NTD-1/2TMD (6.7%) with LIC12757 in the log phase were statistically indistinguishable from the negative control, indicating no significant binding under these conditions (Figure 2B). Notably, the interaction strength of NTD-TMD was comparable to that of FL LIC12756, suggesting that under optimal bacterial growth conditions in the log phase, the ECR is dispensable or functionally inactive, and that the full TMD is sufficient to reinforce the NTD–LIC12757 interaction.

In the stationary phase, all variants, including NTD (79.5%), exhibited measurable interactions of varying strength, suggesting that growth-phase-dependent physiological changes or additional bacterial cellular factors may modulate these interactions (Figure 2B). Notably, NTD alone displayed stronger interaction with LIC12757 than the NTD-1/2TMD variant (48.6%) but weaker than the full NTD-TMD variant (102.7%). This difference likely reflects a more accessible binding interface and preserved native NTD conformation, whereas the partial TMD fragment may sterically hinder or alter the domain’s structure, thereby interfering with the NTD–LIC12757 interaction. Importantly, during the stationary phase, the strongest interaction was observed in the presence of FL LIC12756 (140%), suggesting that the ECR may enable the NTD to sense and respond to environmental and growth-phase-dependent signals, allowing full regulatory activity of LIC12756 toward LIC12757.

**Figure 3:**
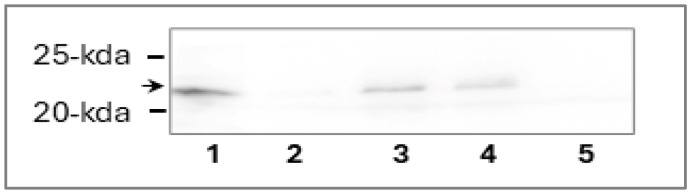
Pull-down analysis of the interaction between LIC12756 NTD and LIC12757. His_6_-tagged NTD of LIC12756 immobilized on Ni-NTA agarose beads was incubated with LIC12757-containing lysate. After washing with 20 mM imidazole, bound proteins were eluted with 250 mM imidazole in three consecutive fractions (Elution 1–3, lanes 2–4). Lane 5 shows the background control sample obtained from Ni-NTA beads without immobilized NTD. Proteins were analyzed by SDS–PAGE followed by ECL Western blotting with anti-LIC12757 serum. The gel was cut at the 35 kDa protein marker before protein transfer onto the nitrocellulose membrane. Therefore, a fragment of the membrane with protein markers below 35 kDa is shown; no higher molecular weight non-specific bands were observed, confirming antibody specificity under these experimental conditions. Lane 1 shows LIC12757 (~21 kDa) present in the lysate used for the pull-down assay (input control), and its position is indicated by the arrow on the left. Positions of the 25- and 20 kDa protein markers (Perfect Tricolor Protein Ladder 11–245 kDa, EURx, Gdańsk, Poland) are also shown on the left.

Direct binding of NTD to LIC12757 was also tested using a pull-down assay with His_6_-tagged NTD (LIC12756) overproduced in the *E. coli* pET system (see Section 2, Section 2.2 and Section 2.3.2). Binding of His_6_-tagged NTD to Ni-NTA agarose beads was confirmed by Coomassie-stained gel and Western blot (Figure S1A,B), while Figure 3 (lane 1) confirms the presence of LIC12757 in the lysate used in this assay. The agarose resin without immobilized NTD was used as a control to exclude non-specific binding of LIC12757 (Figure 3, lane 5). As shown in Figure 3 (lanes 3 and 4), Western blot analysis using specific anti-LIC12757 antibodies detected LIC12757 in the pulled-down fractions (Figure 3, lanes 3 and 4), confirming a direct interaction between the NTD of LIC12756 and LIC12757.

Taken together, these results highlight the NTD as the primary mediator of the interaction between LIC12756 and LIC12757, while the TMD and ECR modulate interaction strength in response to cellular and environmental factors associated with the growth phase.

### 3.2. Effect of LIC_12756 Domains on LIC12757 Transcriptional Activity

To explore the contribution of specific LIC12756 domains to the regulation of LIC12757 transcriptional activity, a previously described *prLIC_12757-luxAB* transcriptional fusion was used [34], whose activity is positively autoregulated [34]. This reporter was combined with plasmids expressing *LIC12757* together with different *LIC12756* variants (see Section 2 and Section 2.4 for details). Of note, in this experiment, the luciferase activity reflected *prLIC_12757* promoter activity, which corresponds to transcriptional activity of LIC_12757. To verify that the T18-NTD fusion (pUT18C) does not affect NTD functionality, a control plasmid encoding LIC12757 together with NTD LIC12756 (pAC7) was generated using a PCR-based method described in Section 2 (Section 2.2), with the primers listed in Table 2. Measurements of *prLIC12757* activity confirmed that the unmodified NTD retained anti-σ activity without exhibiting pro-σ activity, validating that the fusion does not interfere with NTD functionality (Figure S2).

**Figure 4:**
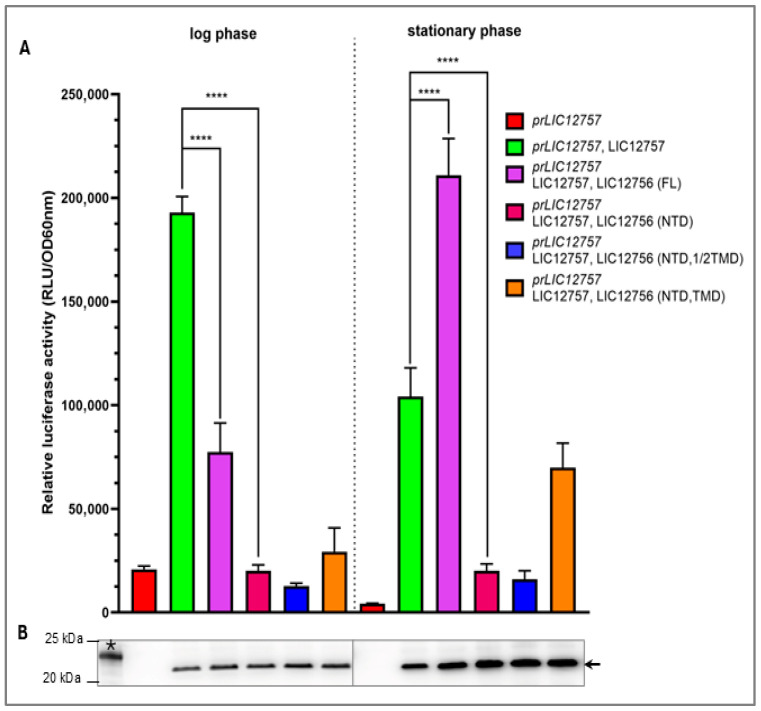
Effect of LIC12756 variants on the *LIC12757* promoter activity. (**A**) Luciferase reporter assay showing the activity of *prLIC12757* in the absence or presence of LIC12757 alone and different variants of LIC12756 (NTD, NTD-1/2TMD, NTD-TMD, FL) during log (log; OD_600_ ~ 0.7–0.8) and stationary (OD_600_ ~ 1.5–1.6) phases. *E. coli* MG1655 cells were transformed with *prLIC12757luxAB* alone (control 1), with *prLIC12757luxAB* and pAC-LIC12757 (control 2), or with *prLIC12757luxAB*, pAC-LIC12757 and the indicated pUT18C-LIC12756 variant. Cultures were grown in LB with appropriate antibiotics and inducers (IPTG 0.5 mM, arabinose 0.02%) at 30 °C. Luciferase activity was measured and reported as relative luminescence units (RLU) normalized to OD_600_. Results represent the mean of three independent experiments, each performed with duplicate cultures, with standard deviations indicated. Statistical analysis was performed using an unpaired two-tailed *t*-test with Welch’s correction (GraphPad Prism 10). All comparisons between LIC12757 alone and LIC12756 variants were statistically significant (**** *p* < 0.0001), although only selected comparisons are shown for clarity. The dashed line indicates the log–stationary phase boundary. (**B**) ECL Western blot analysis of LIC12757 level in cells producing full-length LIC12756 (FL) and its truncated variant. Total cell lysates from logarithmic and stationary cultures, collected prior to promoter activity assay, were loaded in equal amounts and detected with anti-LIC12757 antibody. The gels were cut below the 35 kDa protein marker before protein transfer onto the nitrocellulose membrane. Therefore, only the 20 and 25 kDa markers (from Perfect Tricolor Protein Ladder 11–245 kDa, EURx, Gdańsk, Poland) are shown on the left. The position of LIC12757 (~21 kDa) is indicated by an arrow, and purified His_6_-tagged LIC12757 (~23 kDa; used as a control) [35] is marked with an asterisk.

As shown in Figure 4, during log-phase growth, FL LIC12756 inhibited LIC12757-dependent transcription, as indicated by lower *prLIC_12757* activity compared to cells producing LIC12757 alone, confirming its proposed anti-σ function [35].

Notably, the truncated LIC12756 variants, including NTD, NTD-1/2TMD, and NTD-TMD, exerted even stronger inhibitory effects, indicating that NTD is sufficient for LIC12757 inhibition and promoter repression under optimal growth conditions. In contrast, during the stationary phase, when environmental conditions change and stress signals appear, FL LIC12756 promoted LIC12757 activation, as indicated by the increase in *prLIC_12757* activity (Figure 4A). The truncated variants of LIC12756 failed to stimulate LIC12757 activation, although the NTD-TMD variant displayed slightly higher *prLIC12757* activity compared to the log phase. However, its activity remained significantly lower than that observed in cells expressing LIC12757 alone (Figure 4A), highlighting the role of the ECR in enabling full transcriptional activation under stationary-phase conditions.

Next, to ensure that the observed differences in *prLIC12757* activity were not due to variations in LIC12757 protein levels, Western blotting using an anti-LIC12757 antibody was performed (Figure 4B). This analysis confirmed that differences in *prLIC12757* activity do not result from changes in LIC12757 abundance across the tested cells. Interestingly, in the stationary phase, the LIC12757 level was markedly elevated in the presence of FL LIC12756 and its truncated variants but was comparable across all variants. This suggests that the NTD alone is sufficient to stabilize LIC12757 and probably protects it from proteolysis, as previously proposed for FL LIC12756 [35].

In summary, these results demonstrate that the N-terminal domain of LIC12756 functions as an anti-σ domain toward LIC12757 and simultaneously stabilizes this σ factor. TMD stabilizes NTD, reinforcing the interaction with LIC12757. Full-length LIC12756, which also includes the C-terminal extracytoplasmic region, is required for full transcriptional regulation. The regulatory effects are growth-phase-dependent: during logarithmic growth, LIC12756 primarily suppresses LIC12757, whereas in the stationary phase, full-length LIC12756 enables its activation, which is consistent with its previously proposed dual regulatory role [35]. Furthermore, these results suggest that full activation of LIC12757 likely requires additional extrinsic signals as well as proteolytic processing.

## 4. Discussion

This study provides new insights into the regulatory mechanism controlling the activity of an ECF σ factor LIC12757 from the pathogenic spirochaete *L. interrogans.* The results support previous observations that LIC12756 acts as a dual regulator [35] whose activity depends on both domain organization and bacterial growth phase. It was demonstrated here that the N-terminal cytoplasmic domain of LIC12756 functions as the core anti-σ module responsible for inhibiting LIC12757 (Figure 4A) and serves as the primary domain mediating direct interaction with LIC12757 (Figure 2 and Figure 3). In addition, as shown in Figure 4B, NTD alone appears sufficient to stabilize LIC12757, suggesting that this domain contributes to protecting this σ factor from proteolysis, which is consistent with earlier observations for full-length LIC12756 [35]. In turn, the transmembrane domain and ECR (C-terminal periplasmic domain) are likely to modulate LIC12756’s regulatory activity and enable full activation of LIC12757 under specific physiological conditions. Notably, the ECR domain is likely responsible for sensing environmental signals, which is consistent with the organization of the FecR-like proteins [17,20,21,44].

A striking observation is that NTD and NTD-1/2TMD fail to interact with LIC12757 during logarithmic growth but display such interaction in the stationary phase (Figure 2B). Based on combined results from the BACTH and in vivo promoter activity assays, these observations can be explained by the likely unstable nature of these interactions. Such instability may occur under optimal, non-stressed conditions, when LIC12757 is not yet required. Recent studies have shown that the activation of LIC12757 occurs primarily under stress conditions, such as iron deficiency and nutrient limitation, during the stationary phase [35]. In contrast, NTD-TMD exhibits measurable and comparably strong interactions with FL LIC12756 even in the logarithmic phase, suggesting that membrane anchoring via TMD may stabilize the interaction of NTD with LIC12757 during logarithmic growth. The in vivo promoter activity assay further indicates that the presence of a LIC12757-dependent promoter may contribute to stabilizing these interactions (Figure 4A). This may explain why *prLIC12757* shows very low activity despite LIC12757’s presence in the log phase when the truncated LIC12756 variants are simultaneously present.

Taken together, these findings suggest that the apparent differences in the strength and detectability of interactions between LIC12756 variants with LIC12757 very likely reflect a physiological regulation. This regulation depends on growth phase, environmental conditions, and the presence of stabilizing elements, such as the relevant promoter and membrane anchoring.

Based on these findings, LIC12756 appears to integrate its intrinsic structural features with environmental cues to fine-tune LIC12757-dependent transcription. In the log phase, NTD stabilized by TMD mediates efficient inhibition of LIC12757 activity. In contrast, during the stationary phase, the ECR present in the full-length protein likely facilitates sensing of environmental or stress-related signals, leading to LIC12757 activation and subsequent LIC12757-dependent transcription.

Further studies are required to identify the specific signals and molecular mechanisms responsible for this regulatory switch, in addition to the previously suggested iron and nutrient limitations [35]. These may include host-associated factors, proteolytic processing, and additional cellular components involved in LIC12756 activation. Notably, in *E. coli*, the S2P intramembrane protease RseP plays a critical role in sequential proteolysis of the anti-σ regulator FecR, generating the N-terminal cytoplasmic fragment required for activation of its cognate σ factor FecI and the transcriptional upregulation of the ferric citrate uptake operon [20,21]. Whether a similar regulated intramembrane proteolysis contributes to LIC12756 activation in *L. interrogans* remains to be investigated.

Comparative genomic analyses indicate that *L. interrogans* encodes multiple TonB-dependent receptors (~12 genes) involved in nutrient uptake [45]. However, none of them display the characteristic N-terminal extensions required to directly transmit signals to anti-σ factors, as observed for the *E. coli* FecI-FecR system. The potential involvement of additional surface-associated components, such as TonB-dependent receptors, may expand the responsiveness of this system to diverse environmental or host-derived signals. Whether any of these receptors indirectly modulate LIC12756–LIC12757 remains to be determined.

**Figure 5:**
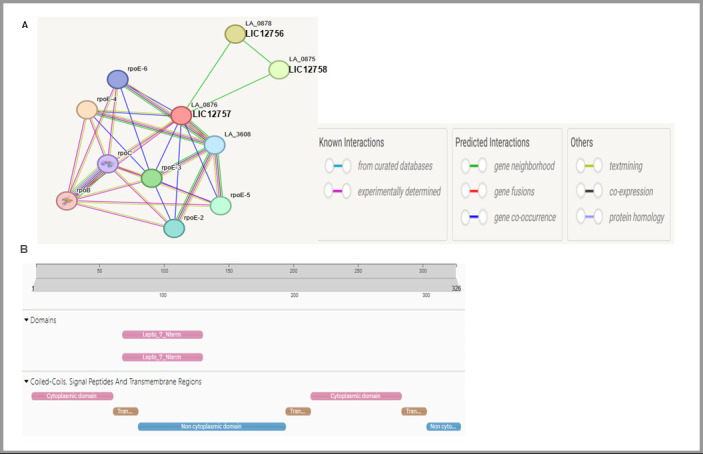
Potential role of LIC12758 (Hsa-like protein) in the LIC12756–LIC12757 regulatory network. (**A**) STRING analysis was performed using the *L. interrogans* serovar Lai database. These results suggest potential functional associations of LIC12758 with LIC12756 and LIC12757, highlighting possible links within this regulatory network. Differences in gene annotations arise from the STRING database being based on the Lai serovar, with the corresponding *L. interrogans* Copenhageni genes also indicated. (**B**) InterPro domain analysis [46] shows that LIC12758 is a membrane-associated protein with a Lepto_7 N-terminal domain (upper panel, pink) and several transmembrane segments (brown; Tran...) spanning cytoplasmic (lower panel, pink) and extracellular regions (blue), which may facilitate environmental sensing or host interactions. The protein sequence was retrieved from GenBank (accession number AAS71315.1).

Interestingly, preliminary in silico analysis using the STRING platform (https://string-db.org/), based on the Lai serovar (Figure 5A), suggests a potential functional link between LIC12758 (Hsa-like protein) and the LIC12756–LIC12757 regulatory system. Domain prediction using InterPro software [46] indicates that LIC12758 is a membrane-associated, surface-exposed protein that could participate in host interactions or environmental sensing (Figure 5B). These analyses are hypothesis-generating, and future studies are required to validate these findings and clarify the role of LIC12758 within this regulatory pathway. Elucidating these interactions may provide important insight into how this system functions as a regulatory and signaling network that enables *L. interrogans* to adapt to environmental stresses, support its survival, and contribute to the pathogenesis of leptospirosis.

## 5. Conclusions

The results of this study support the role of LIC12756 as a dual regulator of the ECF σ factor LIC12757 in the pathogenic spirochaete *L. interrogans*, with its activity modulated by domain organization and bacterial growth phase. The N-terminal cytoplasmic domain mediates anti-σ activity, whereas the transmembrane and ECR domains enable full activation of its σ partner in response to environmental or stress-related signals, which is consistent with features of FecR-like proteins. Furthermore, analyses mentioned in Section 4 suggest that LIC12758 (Hsa-like protein) may represent a potential component of this regulatory network. Elucidating the molecular mechanisms governing this system will improve our understanding of how *L. interrogans* coordinates stress adaptation with pathogenicity.

## Figures and Tables

**Table 2:** Oligonucleotides used for PCR amplification. DNA primers were obtained from Eurofins Genomics (Ebersberg, Germany). Restriction sites are underlined; the arrow indicates subcloning in the BACTH system vector or the pET system.

Oligonucleotide	Sequence (5′ to 3′)	Purpose
pF12756PstI	CTGCAGGATGGAACCTAATTCAGATTC	cloning of *LIC12756* fragments into pJET1.2/blunt → pUT18C
Prev219_12756KpnI	GGTACCCGTGAATTTCTAATATAGAAAG	cloning of a 219 bp fragment of *LIC12756* into pJET1.2/blunt → pUT18C
Prev249_12756KpnI	GGTACCCGAAGCAAAAGAACACAAGCAG	cloning of a 249 bp fragment of *LIC12756* into pJET1.2/blunt → pUT18C
Prev279_12756KpnI	GGTACCCGAAAAAATCTGAAATAAAAA	cloning of a 279 bp fragment of *LIC12756* into pJET1.2/blunt → pUT18C
pFLIC12756NdeI	CATATGGAACCTAATTCAGATTCAAATCG	cloning of a 219 bp fragmet of *LIC12756* into pJET1.2/blunt →pET28
prev219_LIC12756HindIII	AAGCTTATGAATTTCTAATATAGAAAG	cloning of a 219 bp fragmet of *LIC12756* into pJET1.2/blunt →pET28 and together with *LIC12757* into pAC7
pF12757NdeI	CATATGGCCAAAATTCCGAAAG	cloning of *LIC12757* with *LIC12756* 219 bp fragment into pAC7

## Data Availability

Data will be made available on request.

## Supplementary Materials

The following supporting information can be downloaded at: https://www.mdpi.com/article/10.3390/pathogens15040379/s1, Figure S1: Verification of His_6_-tagged NTD (LIC12756) binding to Ni-NTA agarose beads; Figure S2: Effect of the unmodified N-terminal domain (NTD) of LIC12756 on *prLIC12757* activity; Table S1: Mean values of β-galactosidase activity (in Miller units/mL) in the BACTH system; Table S2: Exact *p*-values for β-galactosidase activity comparisons.

## Abbreviations

The following abbreviations are used in this manuscript:

NTD	N-terminal domain
log	logarithmic/exponential phase of growth
TMD	Transmembrane domain
ECF	Extracytoplasmic function
ECFs	ECF σ factors
ECR	C-terminal non-cytoplasmic region/extracellular region
