# Supplementary material for: The N-Terminal Domain of LIC12756 Is the Key Determinant of This Protein’s Anti-Sigma Activity Toward LIC12757 in Pathogenic Leptospira interrogans
Source: Pathogens. 2026 Apr 1;15(4):379. doi: 10.3390/pathogens15040379 (PMC13118517; doi:10.3390/pathogens15040379)
Supplement: Supplementary file 1 [file pathogens-15-00379-s001.zip › pathogens-4226228-supplementary.pdf]

Supplementary Materials

The N-Terminal Domain of LIC12756 Is the Key Determinant of This Protein's Anti-Sigma Activity Toward LIC12757 in Pathogenic *Leptospira interrogans*

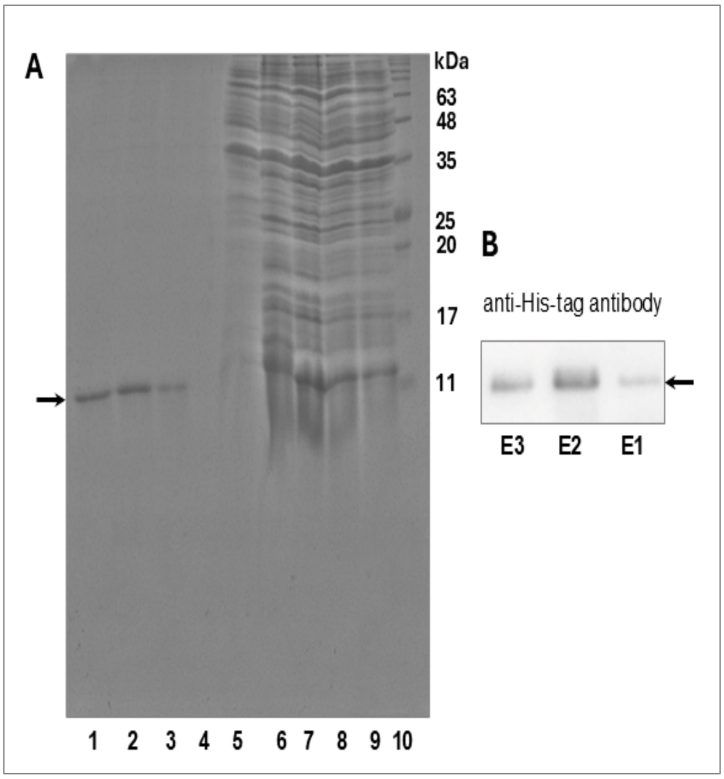

Figure S1. Verification of His_6-tagged NTD (LIC12756) binding to Ni-NTA agarose beads. Coomassie-stained 0.1%SDS–18%PAGE (**A**) and Western blot (**B**) show His_6-tagged NTD overproduced in *E. coli* BL21 (λDE3) strain. Cells were induced with 0.4 mM IPTG at 37 °C for 3 h (used for Ni-NTA binding); overproduction of NTD at 16 °C for ~20 h was also tested but not used for binding. Lanes: 1–3, eluates E3–E1; 4, last wash (fraction obtained after washing the Ni-NTA resin with 20 mM imidazole buffer); 5, bacterial lysate after binding to Ni-NTA agarose; 6, 37 °C cell lysate (tested for expression and pull-down assay); 7, 16 °C cell lysate (tested for expression only); 8–9, 37 °C lysate used for binding; 10, protein markers (Perfect Tricolor Protein Ladder, 11–245 kDa, EURx, Poland). His-tagged NTD (~11 kDa) is indicated by an arrow. Western blot was performed using anti-His tag antibody (1:500; Cell Signaling Technology) and HRP-conjugated goat anti-rabbit IgG (1:3000; Abcam), developed with BioVision ECF substrates (Gentaur, Poland), and imaged on an Azure imaging system (Azure Biosystems, Dublin, CA, USA). The gel was cut at the 17 kDa protein marker before protein transfer onto the nitrocellulose membrane. Therefore, a fragment of the membrane with 11- kDa protein marker below is shown. The position of NTD LIC12756 (~11 kDa) is indicated by an arrow on the right; E3–E1, eluates.

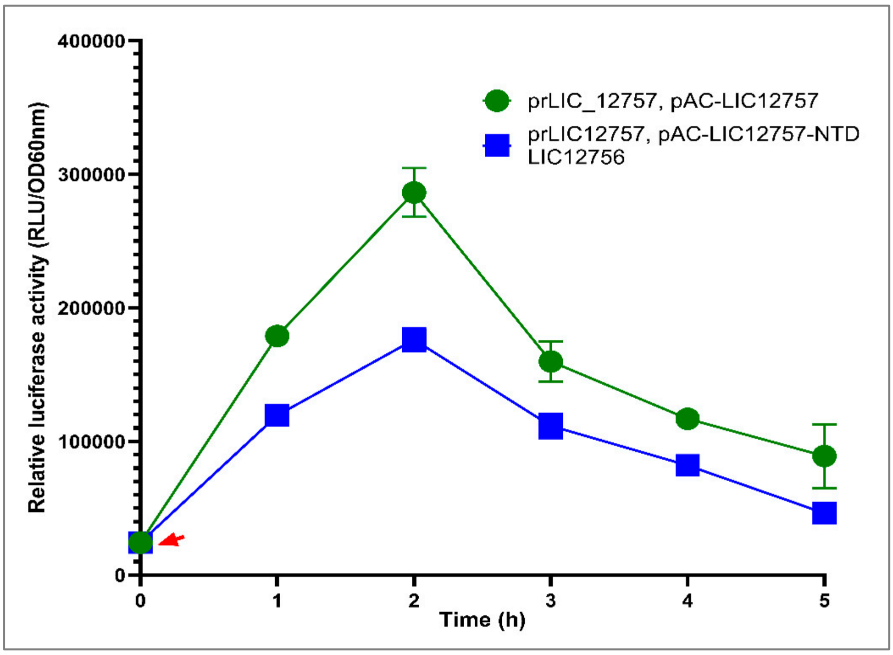

Figure S2. Effect of the unmodified N-terminal domain (NTD) of LIC12756 on *prLIC12757* activity. Luciferase reporter assay showing the activity of *prLIC12757* in the presence of LIC12757 alone or together with the NTD of LIC12756. *E. coli* MG1655 cells were transformed with *prLIC12757luxAB* and pAC-LIC12757, or with *prLIC12757luxAB* and pAC-LIC12757-NTD LIC12756. Cultures were grown at 37 °C in LB supplemented with chloramphenicol (40 µg/ml) and spectinomycin (65 µg/ml) to an OD_{600} of ~0.43-0.45 and arabinose (0.02%) was then added to induce the protein production. Samples were collected at the indicated time points. Luciferase activity was measured and reported as relative luminescence units (RLU) normalized to OD_{600}. The results are presented as the average of three independent experiments performed in duplicate, and error bars indicate standard deviation (SD). The red arrow indicates the time of arabinose addition.

Tabel S1. Mean values of β-galactosidase activity (in Miller units/ml) in the BACTH system

**Sample**	**β-galactosidase activity (U/ml) (average of three independent experiments)**
	**Log phase**
Negative control	545.9
Positive control	6390
T-25-LIC12757, T18-LIC12756 FL	4984
T-25-LIC12757, T18-LIC12756 NTD	575.7
T-25-LIC12757, T18-LIC12756 NTD-1/2TMD	424.9
T-25-LIC12757, T18-LIC12756 NTD-TMD	5375
	**Stationary phase**
Negative control	309.8
Positive control	6471
T-25-LIC12757, T18-LIC12756 FL	9057
T-25-LIC12757, T18-LIC12756 NTD	5146
T-25-LIC12757, T18-LIC12756 NTD-1/2TMD	3143
T-25-LIC12757, T18-LIC12756 NTD-TMD	6648

Table S2. Exact p-values for β-galactosidase activity comparisons

Comparison	p-values
**Log phase**	
Negative control vs T-25-LIC12757, T18-LIC12756 FL	p < 0.0001 (****)
Negative control vs T-25-LIC12757, T18-LIC12756 NTD	p= 0.0899 (ns)
Negative control **vs** T-25-LIC12757, T18-LIC12756 NTD-1/2TMD	p= 0.3016 (ns)
Negative control vs T-25-LIC12757, T18-LIC12756 NTD-TMD	p < 0.0001 (****)
Positive control vs T-25-LIC12757, T18-LIC12756 FL	p= 0.0030 (**)
Positive control vs T-25-LIC12757, T18-LIC12756 NTD	p < 0.0001 (****)
Positive control vs T-25-LIC12757, T18-LIC12756 NTD-1/2TMD	p < 0.0001 (****)
Positive control vs T-25-LIC12757, T18-LIC12756 NTD-TMD	p= 0.0436 (*)
**Stationary phase**	
Negative control vs T-25-LIC12757, T18-LIC12756 FL	p < 0.0001 (****)
Negative control vs T-25-LIC12757, T18-LIC12756 NTD	p < 0.0001 (****)
Negative control vs T-25-LIC12757, T18-LIC12756 NTD-1/2TMD	p < 0.0001 (****)
Negative control vs T-25-LIC12757, T18-LIC12756 NTD-TMD	p = 0.0022 (**)
Positive control vs T-25-LIC12757, T18-LIC12756 FL	p < 0.0001 (****)
Positive control vs T-25-LIC12757, T18-LIC12756 NTD	p= 0.0230 (*)
Positive control vs T-25-LIC12757, T18-LIC12756 NTD-1/2TMD	p= 0.0012 (**)
Positive control vs T-25-LIC12757, T18-LIC12756 NTD-TMD	p= 0.2962 (ns)
